# Oral Health in Patients with History of Head and Neck Cancer: Complexity and Benefits of a Targeted Oral Healthcare Pathway

**DOI:** 10.1007/s11912-024-01507-8

**Published:** 2024-02-20

**Authors:** Marion Florimond, Lucas T. Duong, Elodie Lours, Jean-Jacques Brau, François C. Ferré, Isabelle Fouilloux, Tchilalo Boukpessi

**Affiliations:** 1 URP 2496 BRIO, Biomedical Research in Odontology, Université Paris Cité, 1 Rue Maurice Arnoux, 92120 Montrouge, France; 2 Dental Faculty, Department of Oral Biology, Université Paris Cité, Paris, France; 3 Dental Department, Charles Foix Hospital, AP-HP, 94200 Ivry Sur Seine, France; 4 Centre de Recherche Des Cordeliers, UMRS 1138, Molecular Oral Pathophysiology, Université Paris Cité, INSERM, Sorbonne Université, Paris, France; 5 Dental Faculty, Department of Oral Surgery, Université Paris Cité, Paris, France; 6 Department of Head and Neck Surgical Oncology, Institut Gustave Roussy, Villejuif, France; 7 Dental Faculty, Department of Prosthetics, Université Paris Cité, Paris, France; 8 Dental Department, Pitié Salpêtrière Hospital, AP-HP, 75013 Paris, France; 9 Dental Faculty, Department of Restorative Dentistry and Endodontics, Université Paris Cité, Paris, France

**Keywords:** Head and neck neoplasms, Radiotherapy, Oral health, Dental care, Quality of life, Critical pathways

## Abstract

**Purpose of Review:**

This work consists in a literature review on the current state of knowledge regarding the oral management of patients with a history of head and neck cancer (HNC), corroborated by clinical cases and illustrated by clear infographic summaries. It aims to provide healthcare professionals with a comprehensive overview of the oral health status of HCN patients.

**Recent Findings:**

Head and neck cancers (HNCs) represent the seventh most common type of cancer worldwide, with over 660,000 annual new cases. Despite the significant negative impact of HNCs on oral health, patients often receive no or inappropriate oral care while the significant impact of oral pathologies on cancer prognosis is commonly underestimated.

**Summary:**

This work (i) describes the oral cavity during and after HNC through the prism of care complexity and (ii) highlights several potential key factors that could worsen long-time patients’ prognosis and quality of life. By investigating the biological, microbiological, functional, and psychological dimensions of the interrelationships between HNCs and oral health, the authors explored the barriers and benefits of a targeted oral healthcare pathway. This article emphasizes the importance of multidisciplinary care and highlights the need for further research elucidating the intricate relationships between oral health and HNCs, particularly through the microbiota.

## Introduction

Head and neck cancers (HNCs) include a wide variety of malignant pathologies arising from the oral cavity, nasopharynx, oropharynx, larynx, and hypopharynx. Although 90% of them are squamous-cell carcinomas affecting mucosal surfaces, they are highly heterogeneous in their epidemiology, pathophysiology, and treatment [1, 2]. They represent the seventh most common type of cancer worldwide, and the World Health Organization (WHO) estimated that 439,000 cases of oral and oropharyngeal cancer would be diagnosed by 2030 [3]. The current therapeutic arsenal includes surgery, radiotherapy, chemotherapy, targeted therapy, and immunotherapy. Therapeutic decision of HNC is based on a multidisciplinary assessment of the patient’s performance status, the disease’s status, the anatomical situation, and the complexity of a surgical approach. Most of the treatment options, often used in combination, have significant side effects that can lead to long-term sequelae [4–6]. Even though the last few decades have seen an improvement in management, with an increase in survival rate and quality of life, patients with a history of HNC (HNC patients) constitute a population with specific needs throughout their life course, particularly with regard to oral health. The aim of this work is therefore to provide a comprehensive overview of the oral health status of HNC patients, targeted at every actor in the healthcare pathway. This literature review provides (i) an in-depth description of the oral cavity during and after HNC through the prism of care complexity and (ii) a highlight of several potential key factors that could worsen long-time patients’ prognosis and quality of life.

## Carious Disease in HNC Patients

### Pathogenesis and Epidemiology

**Fig. 1:**
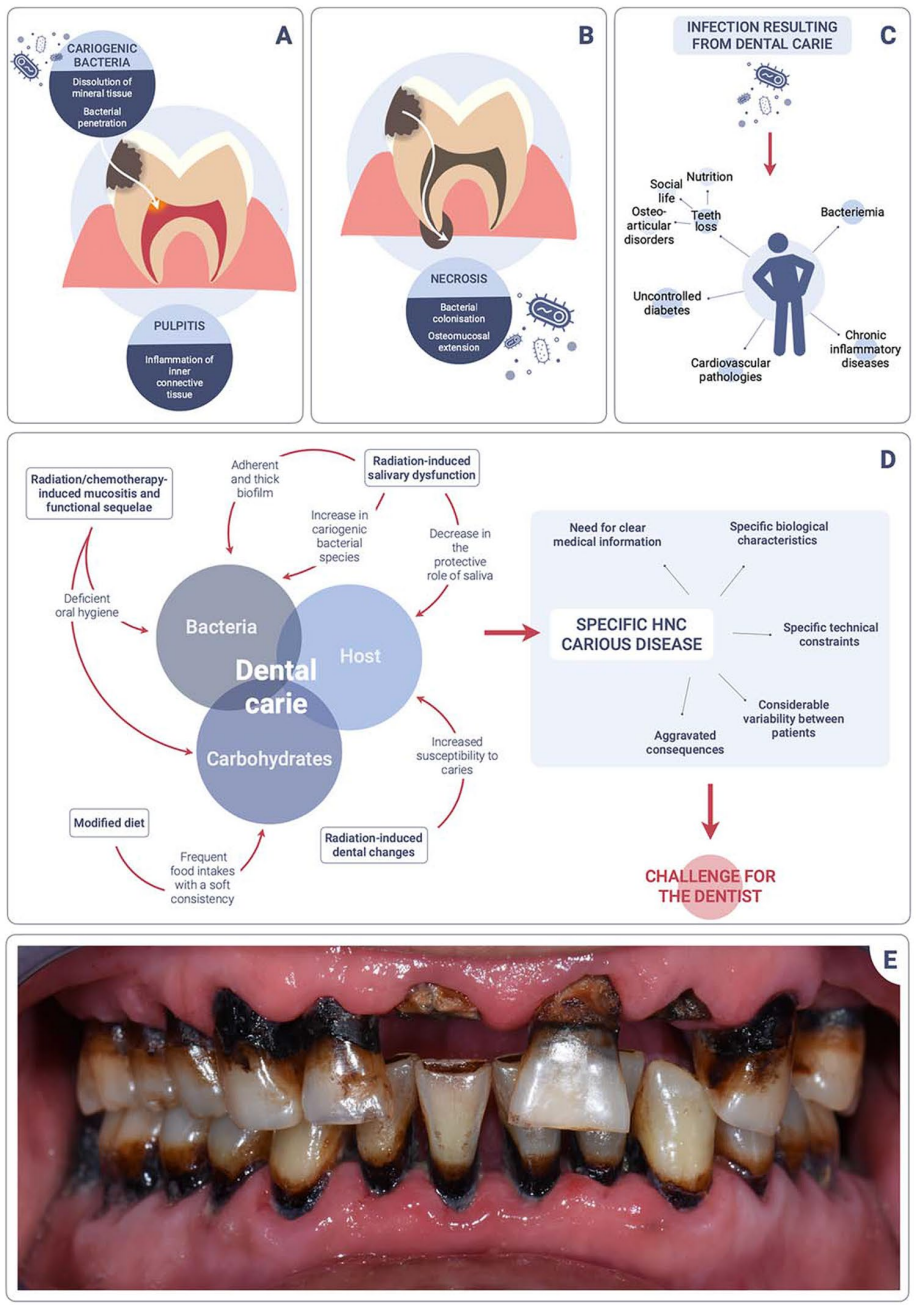
Carious disease in HNC patients. **A** Initiation of the carious process leading to pulpitis. **B** Progression of the carious process until pulp necrosis. **C** Systemic impact of carious disease. **D** Specific features of HNC carious disease. **E** Clinical features of HNC carious disease. Patient in remission from undifferentiated carcinoma of nasopharyngeal tumor (UCNT) treated in 2015 in Tunisia with radiochemotherapy. The dose received is estimated at 50 Gy for the teeth in the posterior sectors and 35 Gy for the teeth in the anterior sectors. No dental treatment was ever provided. The carious lesions are typical of those described in the literature in HNC patients. Dark cervical lesions, which progress centripetally until tooth fracture, are observed

Carious disease is an infectious pathology characterized by the destruction of dental tissue by acidogenic bacteria that metabolize carbohydrates from the diet [7]. These microorganisms belong to a cariogenic biofilm which develops in a context of dysbiosis of the oral ecosystem. All too often regarded as inevitable, harmless, and without significant consequences, the carious disease constitutes a major public health problem with significant economic cost [8]. It is considered by the WHO to be the fourth most costly chronic pathology, affecting more than 2 billion people worldwide and consuming over 10% of healthcare budgets in industrialized countries [9, 10]. It has also been established that the consequences of dental caries can have systemic implications. Tooth decay first leads to pulpitis, a painful inflammation of the inner connective tissue (Fig. 1A). If left untreated, pulp tissue necroses and becomes colonized by bacteria, which then spread to the underlying bone (Fig. 1B). Dental caries can lead to tooth loss, with a negative impact on nutrition, social life, and the osteoarticular system. In addition, the bacteriemia resulting from dental infection can trigger a pro-inflammatory syndrome, playing a role in uncontrolled diabetes, the pathogenesis of cardiovascular pathologies, and the evolution of chronic inflammatory diseases [11, 12•, 13] (Fig. 1C).

### Carbohydrates

Carbohydrate intake, an essential factor in the pathogenicity of cariogenic bacteria which is determined by eating habits, can be influenced in the short and long term in HNC patients. Indeed, patients’ diet may be altered by pain due to tumor growth, mucositis induced by radiotherapy and chemotherapy, chewing disorders following surgery, deglutition disorders requiring adaptation of food texture, treatment-induced dysgeusia, xerostomia, depression, etc. A fractioned diet over the day rich in carbohydrates and of soft consistency promotes carious disease [14]. HNC patients may therefore have increased dietary risk factors, with great individual variability depending on the type of cancer, treatments received, and dietary adaptations.

### Oral Microbiota

The oral microbiota is a pillar of the complex oral ecosystem. Its balance is influenced by a wide range of factors [4, 7, 15]: local hygiene, age, gender, local pathologies, systemic diseases, diet, drug treatments, salivary changes, etc. It has been documented to be influenced by radiotherapy and chemotherapy in HNC [16•, 17, 18]. In particular, an increase in acidogenic and cariogenic bacterial species, such as *Streptococcus mutans* and *Lactobacillus* species, has been demonstrated in the oral flora during and up to 3 months after radiotherapy for HNC [4]. To the authors’ knowledge, there is no existing long-term data on the oral microbiota of HNC patients. However, persistent sequelae (hyposialia, dysphagia, etc.) can be expected to further affect the microbiota after treatment, with an increased risk of caries in the long term.

### Host Characteristics

As explained earlier, the development of carious lesions is linked to the presence of cariogenic bacteria and fermentable nutrients. However, the characteristics of the ecosystem will also play an essential role. Saliva is a key factor in immunity, buffering capacity, glucose clearance, and remineralization of dental tissue. Nevertheless, radiotherapy-induced salivary hypofunction with quantitative and electrolytic changes is observed in 64–91% of HNC patients treated with radiotherapy [19, 20]. In addition, mucositis, surgical sequelae, limited mouth opening, and depression often observed in HNC patients complicate daily oral hygiene. On the other hand, it is now well established that radiotherapy in HNCs has deleterious effects on the mineral and organic components of dental tissue from 30 Gy onwards [21•, 22••, 23]. The overall hardness of the tooth is said to be impaired with an increased susceptibility to caries progression. In addition, radiation hinders the adhesion of biomaterials commonly used in tooth restoration [24].

### Implications for Dentists and Patients

Carious disease in HNC patients requires special management, both in terms of prevention and treatment (Fig. 1D). Dentists should be able to assess and control the specific risk factors, depending on the dose of radiation received, the resulting hyposialia, oral hygiene difficulties, diet, associated comorbidities, etc.

Moreover, owing to all these specificities, HNC patients whose teeth have been affected by radiotherapy develop highly specific carious lesions (Fig. 1E). These have been described since the 1960s [25] and seem to have a different mechanism of evolution than caries in the general population [26]. Treatment, and especially the choice of biomaterials to be used, must be carefully adapted to the patient’s specific needs, which determine the longevity of the treated teeth, with significant long-term implications on oral health. Clear and effective communication with the medical team allows to assess risk factors. In addition, knowledge and experience in the treatment of teeth whose structure is modified by radiotherapy enable early diagnosis and improved prognosis.

## Periodontal Disease in HNC Patients

### Pathogenesis and Epidemiology

**Fig. 2:**
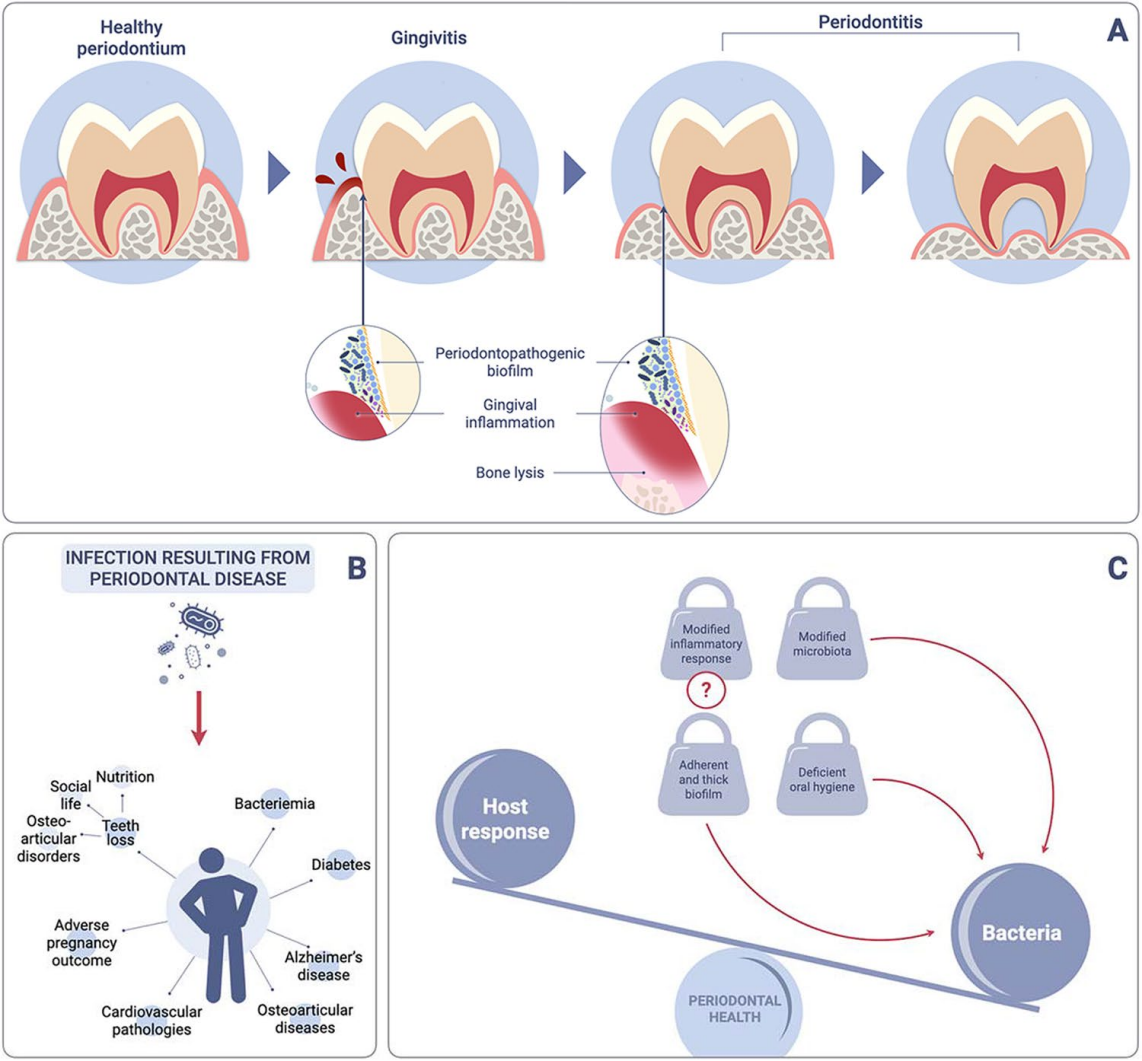
Periodontal disease in HNC patients. **A** Pathophysiology of periodontal disease. **B** Systemic impact of periodontal disease. **C** Specific features of periodontal disease in HNC patients

Periodontal disease is a progressive microbiome-driven inflammatory pathology of the tooth’s supporting structures (gingiva, bone, and periodontal ligament) (Fig. 2A). According to the WHO, over a billion people worldwide suffer from severe periodontal disease [10] which, if left untreated, leads to tooth loss. It constitutes a major public health problem, since in addition to the consequences of edentulism, correlations between periodontal disease and many systemic conditions have been established: cardiovascular diseases, diabetes, adverse pregnancy outcomes, chronic obstructive pulmonary disease, bacterial pneumonia, Alzheimer’s disease, osteoarticular pathologies, etc. [27, 28] (Fig. 2B).

### Bacterial Factor

The bacterial factor is a key element in the pathogenesis of periodontal disease. It relies on the presence of a complex polymicrobial biofilm in dysbiosis [29]. The usual pathogenic elements that have been identified include LPS from gram-negative bacteria, which generates the production of tissue-damaging reactive oxygen species by fibroblasts. However, the proportion of gram-negative species has been shown to increase when oral hygiene was compromised [15], which is common in HNC patients. Moreover, HNCs and treatments may modify the oral microbiota, favoring a periodontopathogenic flora. Some studies, for example, suggested that human *Papillomavirus* positive (HPV +) oropharyngeal cancers were associated with an increase in *Veillonella* species [30]. Interestingly, *Veillonella* species have been described as necessary for the development of *Porphyromonas gingivalis*, a keystone in periodontal disease [31].

However, the oral microbiota associated with HNCs is complex to study, especially as the parameters of the oral ecosystem are highly variable in HNC patients, who present very heterogeneous clinical conditions.

### Host Response

The development and progression of periodontal disease are directly linked to the imbalance between pathogenic bacterial species and host’s defenses. HNC treatments have been shown to impact salivary and tissular levels of pro-inflammatory cytokines such as interleukin-1β (IL-1β) and tumor necrosis factor-α (TNF-α) [32, 33]. Both have been described as potential markers or susceptibility factors for periodontal disease [34]. In addition, myelosuppression is often observed in HNC patients treated with cytotoxic chemotherapy, resulting in an altered immune response [35, 36]. It would therefore appear that periodontal response could be impaired during treatment of HNCs with radiotherapy and chemotherapy. This hypothesis is corroborated by the many studies conducted on the modified host response in the context of mucositis induced by radiotherapy or chemotherapy [32, 33, 37].

### Implications for Patients and Dentists

To the authors’ knowledge, there are currently no studies on the incidence and characteristics of periodontal disease in HNC patients. However, it is possible that oral hygiene issues, combined with changes in the microbiota and immune response, may influence the development and progression of periodontal disease with significant consequences (Fig. 2C). The above-mentioned data therefore suggest that a periodontal treatment, by means of simple yet targeted protocols for controlling dental biofilm, could have significant benefits for HNC patients.

## Prosthetic Rehabilitation in HNC Patients

Prosthetic rehabilitation of HNC patients is a very challenging task [38, 39•, 40] with a major stake as it can significantly impact quality of life [38, 41•, 42•]. As mentioned earlier, tooth restoration requires a specific approach regarding the choice of materials. Indeed, the biological and mechanical changes in dental tissue caused by radiotherapy must be taken into account [24]. Furthermore, replacing missing teeth presents other peculiarities. The tissues supporting removable or implant-supported prostheses can be considerably altered [38, 39•]. In soft tissue, defects, scarring, bulky flaps, and damaged muscle structures may be observed. Changes in bone support may be associated with loss of anatomical structures, sometimes with major aesthetic damage. Therapeutic goals and feasibility are impacted by functional damage such as limited mouth opening, altered lingual function, and impairment of velopharyngeal competence. Moreover, the authors have clinically observed that the subsequent disruption of temporomandibular joint dysfunction and occlusal relations may further complicate the restoration of masticatory function. Yet, studies addressing this topic are lacking.

### Removable Prosthesis

Xerostomia, often present in HNC patients, has been described as an unfavorable factor for the success of removable prostheses. Indeed, it has been suggested that oral dryness may increase pain sensitivity of the oral mucosa in patients with complete removable prostheses [43]. Xerostomia may also have a negative impact on phonation, mastication, and retention in patients with complete or partial removable dentures [44]. Moreover, it would appear that removable dentures associated with xerostomia in HNC patients are more prone to colonization by *Candida* species and therefore to candidiasis [45]. In order to achieve its aesthetic and functional functions, removable prosthetic rehabilitation in HNC patients can only be considered with a thorough understanding of this specific context.

### Implant-Supported Prosthesis

When implant placement is not contraindicated in HNC patients, the specific factors that may affect treatment prognosis must be carefully considered. Despite the lack of any consensus on this subject, some authors suggest that xerostomia could have a negative impact on the prognosis of implant-supported prosthesis by increasing the risk of peri-implantitis [46]. Furthermore, it seems that certain bacterial species associated with peri-implantitis, such as *Prevotella* or *Porphyromonas* species, are increased after radiotherapy in HNC patients [17, 47]. The reported survival rate of dental implants placed in irradiated bone is of 90% after 7 years [48], against 96% after 10 years for the general population [49]. Adequate assessment of therapy’s prognosis therefore depends on the knowledge of HNCs and their potential impact.

### Implications for Patients and Dentists

Prosthetic rehabilitation of HNC patients may involve a large number of difficulties that can considerably vary from one patient to another. Each step can be a challenge and the prognosis can be considerably affected. The objectives of restoring mastication and phonation functions must be patient-specific, while compromises are often bound to occur. The challenges and clinical situations are highly diverse, requiring a great capacity for adaptation. It seems reasonable to think that the quality of prosthetic management of patients could be improved by training dentists in the peculiarities of HNC. Interestingly, one study showed that practitioners’ field of expertise, as well as their type of practice (hospital, university hospital, or private practice), could influence therapeutic decisions regarding prosthetic rehabilitation in HNC patients, especially the choice of a removable or an implant-supported prosthesis [39•], which has a direct impact on patients’ quality of life [42•]. The authors highlighted the benefits of multidisciplinary structures where patients could receive specialized prosthetic care, as well as the need to establish clear treatment guidelines.

## Osteoradionecrosis

### Pathogenesis and Epidemiology

Among all the specific features of HNC patients, osteoradionecrosis (ORN) is one of the most widely described. The rate of ORN is currently estimated at around 5% in HNC patients treated with radiotherapy [5, 50, 51•]. There is currently no consensus on the pathogenesis of ORN, but the hypovascularization resulting from radiation-induced arteritis may play a major role. However, microorganisms, hypoxia, hypocellularity, and altered fibrosis have also been described. The mandible is 6 times more affected than the maxilla [51•] and the consequences can be very disabling, with a major impact on quality of life [52]. In addition to the functional and aesthetic consequences for the patient, the economic cost of ORN is substantial, estimated at up to $74,000 per patient for the most extensive surgical procedures [53]. The links between ORN and oral health have been largely described [4].

### Prevention and Early Diagnosis

Diagnosis is essentially based on clinical observation, since ORN is defined as bone exposure in an irradiated area persisting for more than 3 months, in the absence of persistence or recurrence of the malignancy [50]. It was suggested that the risk of ORN was correlated with the radiation dose received [51•]. Dental extractions are frequently described as a risk factor for ORN, yet this risk has not been quantified [5, 50, 51•]. ORN that are not associated with dental extractions have been described and some studies suggest that this is the case for the majority of ORN [51•]. Other risk factors remain poorly understood. However, it would appear that oral infections with dental and periodontal origin increase the risk of ORN, as does mucosal trauma associated with dentures [4].

### Implications for Patients and Dentists

**Fig. 3:**
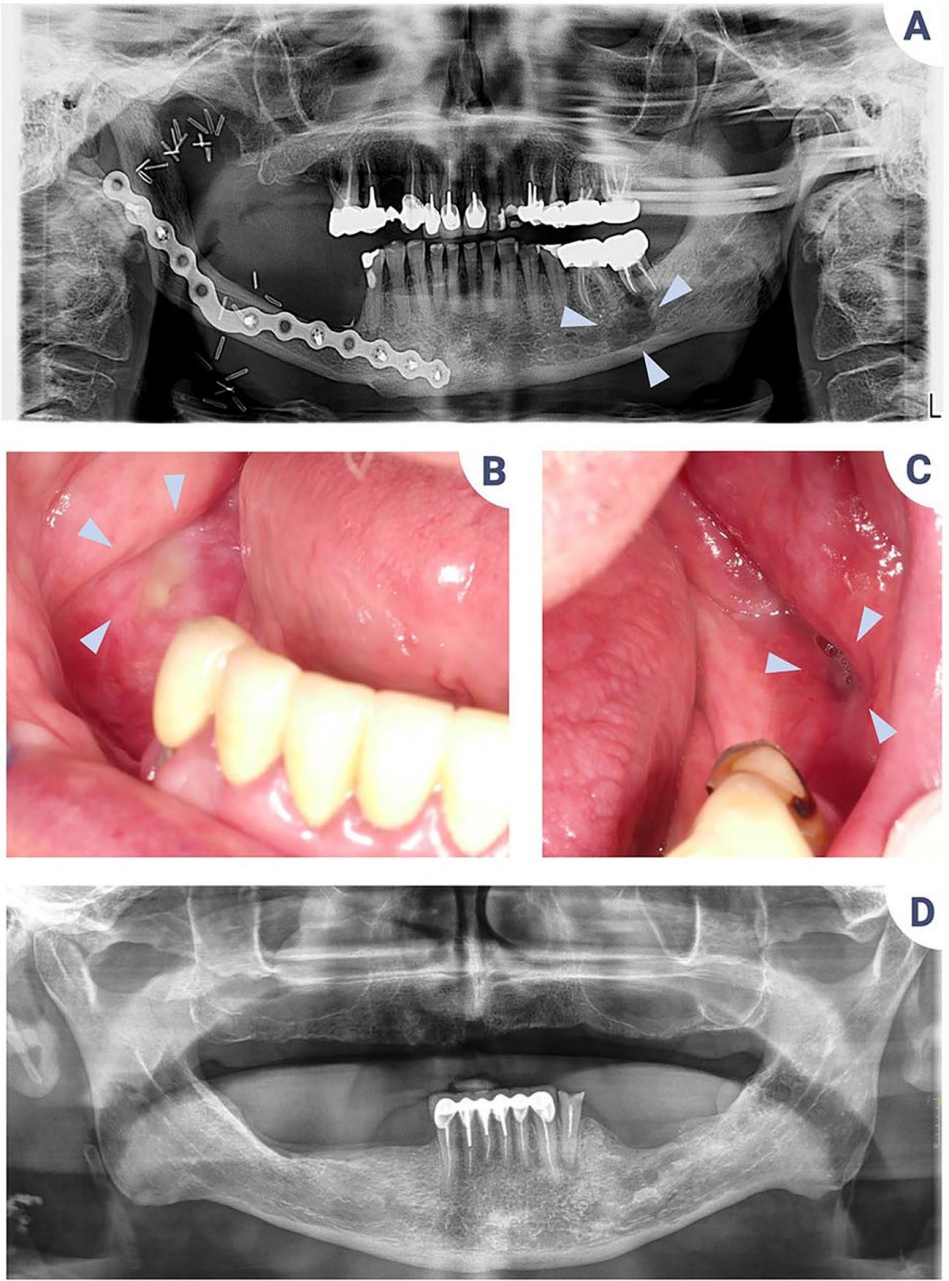
The intricacies of ORN diagnosis. **A** Panoramic X-ray of a patient with acinar cell carcinoma of the right submandibular gland treated in 2000 and recurrences in 2003, 2004, 2012, 2017, and 2020. Treatment consisted in multiple surgeries, radiotherapy, chemotherapy, and proton therapy. The clinical oral examination is complicated by the significant limitation of mouth opening. Tooth no. 37 clinically showed suppuration, suggestive of endodontic or periodontal infection, associated with a periapical radiolucency image (blue arrows). Given the patient’s history, she was referred to the oncology team for an opinion. The diagnosis of ORN was confirmed. **B**–**D** Clinical views and panoramic X-ray of a patient with a history of squamous cell carcinoma of the right tonsil treated by radiochemotherapy in 2007. He presented with two foci of ORN in the posterior mandibular sectors, suggesting traumatic mucosal ulceration due to his removable dental prosthesis. Early diagnosis and drug therapy resulted in stabilization of the situation

Knowledge of the pathology and its consequences is important for optimal prevention and diagnosis. Dentists with experience of HNC patients will be better trained to detect early clinical signs of ORN, which may be too often confused with dental pathologies (Fig. 3). Tooth extractions appear to be a significant risk factor, and numerous surgical protocols for the prevention of ORN have been described in the literature. Although there is no consensus regarding any of them, it is reasonable to consider that extractions should be carried out by dentists or oral surgeons who have experience with HNC patients and who will correctly assess the risks. On the other hand, effective communication with the radiotherapist is essential to know the radiation dose received for each dental sector in order to properly assess the risk of ORN.

## Oral Mucosal Diseases in HNC Patients

### Oral Mucositis

Oral mucositis is estimated to affect 59.4–100% of HNC patients treated with radiotherapy or chemotherapy, with severe forms occurring in 65% of patients [54]. It may have considerable consequences, as it could lead to increased consumption of narcotic analgesics, total parenteral nutrition, interruption of cancer treatment, longer hospital stays, and higher risk of local and systemic infection, with a significant economic cost, estimated at up to $33,560 per patient [17, 55]. Chronic forms of mucositis have been reported after HNC treatment, in the form of atrophic, erythematous, and/or ulcerated lesions [54].

### Oropharyngeal Candidiasis

Oropharyngeal candidiasis is a common condition in HNC patients due to colonization of the oral cavity by *Candida* species that can lead to dysphagia, anorexia, or malnutrition [5, 56]. It has been reported that non-albicans *Candida* species are highly virulent and resistant to antifungal drugs compared to *Candida albicans* and have higher biofilm formation capability. Interestingly, it has been suggested that non-albicans species predominate in HNC patients [57].

### Lichen Planus

A few cases of lichen planus associated with HNCs and their treatments have been reported in the literature [58–60]. In particular, the anti PD-1 (programmed death protein-1) antibody nivolumab, used in the treatment of recurrent or metastatic HNC [61], could be associated with the development of lichen planus. No studies have yet confirmed a correlation between nivolumab and the appearance of lichen planus in HNC patients.

### Implications for Patients and Dentists

These data suggest that ideal oral care of HNC patients requires knowledge of these pathologies in order to diagnose and manage them as effectively as possible. Furthermore, with the advent of novel treatments, such as immunotherapy, new patient profiles may emerge in the coming years. Keeping dentists up to date with advances in cancer research is therefore essential.

## Impact of Oral Health on HNC

### Impact of Microbiota on Recurrence Risk

#### Cariogenic Species

It has been suggested that microbiota may act synergistically with alcohol in carcinogenesis. By metabolizing ethanol, some bacterial species could participate in the formation of acetaldehyde, which increases the risk of HNC by interfering with DNA synthesis and repair [62••]. These bacteria may include *Streptococcus salivarius*, for example, which is associated with oral biofilm formation due to its ability to bind to salivary amylase [63], as well as *Corynebacterium* species, which are involved in caries due to their high saccharolytic and acidogenic properties [64]. These observations may suggest a link between cariogenic flora and the development of alcohol-related HNCs.

#### Periodontopathogenic Species

*Porphyromonas gingivalis*, the keystone of periodontal disease, has been reported to have a potential influence on the development and prognosis of HNCs [65–67]. Indeed, it may increase the migratory and invasive properties of malignant cells and may also enhance matrix metalloproteinase (MMP) secretion [62••, 68]. It has also been shown to induce resistance to Taxol, a chemotherapy medication [68]. *Fusobacterium nucleatum*, another specie associated with periodontal disease, has been suggested to play a role in the genesis of HNCs. It has been suggested that it may be involved in cancer invasiveness, survival, and epithelial-mesenchymal transfer in the oral tumor microenvironment [65].

#### Candida Species

It has been proposed that *Candida* species can produce carcinogenic compounds such as nitrosamines. They could also increase the level of certain MMPs, promote angiogenesis, and have an impact on tumor metabolism, favoring progression and metastatic potential [65, 69].

#### Implications for Patients and Dentists

Understanding of the oral microbiota needs to be improved, but it seems established that pathogens associated with oral pathologies may be linked to the development and progression of HNCs. With an estimated recurrence rate of 17 to 30% [70], it therefore seems essential to make oral health a priority in HNC patients, with potentially positive effects on prognosis and risk of recurrence. By controlling the pathogenic flora, the dentist could play a key role in the prognosis of HNC patients. However, this implies both understanding and knowledge of the impact of oral health in this population in order to provide patients with appropriate care.

### Impact of Oral Health on Osteoradionecrosis and Mucositis

#### Osteoradionecrosis

As mentioned above, oral pathologies increase the risk of ORN, with considerable biological and economic consequences. It was suggested that effective oral management, including prevention of periodontal disease and dental extractions, may reduce the risk of ORN [71]. It has been reported that the incidence of ORN was of 19% in patients with periodontitis. This incidence could be as high as 33% in patients with periodontitis and whose hopeless teeth had not been extracted prior to radiotherapy [72].

Given the major consequences of ORN, restoring and maintaining oral health should be a priority after radiotherapy.

#### Mucositis

In patients with hematologic malignancies, it has been suggested that oral pathologies, particularly periodontal pathologies, affect the severity and duration of mucositis [73••]. Further studies are therefore needed to explore oral pathologies’ potential impact on mucositis in case of HNCs. These data would thus confirm the role of the oral microbiota that has been suggested in the development of mucositis [74]. Some gram-negative bacteria associated with oral pathologies are suspected of being correlated with the severest form of mucositis. For instance, an in vitro study suggested that *Fusobacterium nucleatum*, a periodontopathogenic species associated with severe mucositis, may upregulate some pro-inflammatory cytokines and chemokines, with deleterious effects on epithelial cells [74].

It would therefore seem that controlling periodontopathogenic biofilm during treatment of HNCs could bring major benefits for both patients and healthcare economics.

### Nutrition

The link between weight loss and the prognosis of HNCs has been extensively studied. Weight loss can be caused by mucositis, dysgeusia, xerostomia, and nausea [75]. To the authors’ knowledge, only few studies exist on the reduction in masticatory function in HNC patients and none on the impact of tooth loss on weight in HNC. However, recommendations for the elimination of infectious foci and the prevention of ORN often require multiple tooth extractions prior to radio- and chemotherapy. Cancer removal surgery also sometimes requires dental extractions. It is therefore reasonable to imagine that masticatory function may be impaired at the time of cancer treatment, which may then influence food intake and consequently weight. Existing studies focus mainly on cancers of the oral cavity [76, 77•] or small cohorts [78•]. They highlight a reduction in masticatory performance, which with time could improve in prosthetically rehabilitated patients. Yet, it has been suggested that weight loss is correlated with a poorer HNCs’ prognosis and an increased risk of recurrence [75, 79–81].

**Fig. 4:**
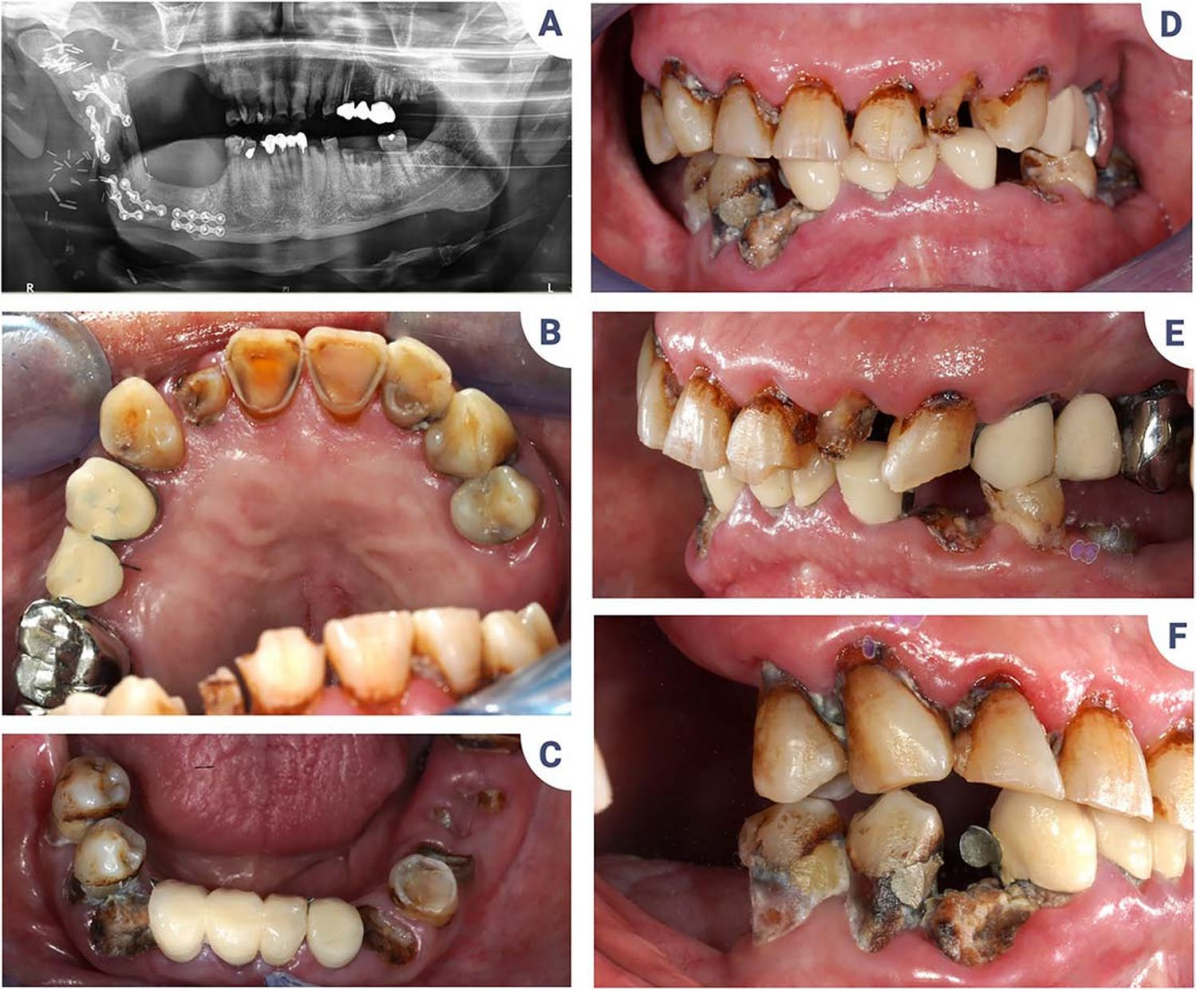
Negative impact of HNCs on nutrition. **A**–**F** Patient in remission from a mandibular squamous cell carcinoma with maxillary extension treated in 2006 by radiotherapy and surgery. Maxillectomy, mandibulectomy, and reconstruction using a free fibula flap left severe functional sequelae. Severe hyposialia and swallowing disorders are also present. Dental treatments must be carried out in a seated position to prevent choking. In the absence of adapted oral care, the situation progressively worsened. In 2021, following the loss of multiple dental restorations, the patient suddenly lost 8 kg as a result of eating difficulties

Given the increase in risk factors and the difficulties in managing them, it is likely that masticatory function may decline after cancer treatment, which may then negatively impact the long-term prognosis (Fig. 4). It is therefore legitimate to suggest that oral pathologies leading to tooth loss may adversely influence the prognosis of HNCs by complicating nutrition and increasing weight loss.

### Anxiety and Depression

It has been suggested that the risk of mortality is increased by 26–39% in patients with a history of cancer and who suffer from a depressive disorder [82]. In addition to its impact on motivation and compliance with treatment, depression causes endocrine and immune changes that could accelerate cancer progression. Interestingly, among the factors associated with anxiety and depressive disorders in HNCs, xerostomia, eating difficulties, and phonation problems appear to play a significant role [83]. There are as of yet no studies on the role of oral pathologies in depressive disorders in HNC patients. However, given that oral cavity diseases have an impact on eating and phonation and that they are aggravated by HNC, they are likely to have a negative impact on the development and progression of depressive disorders, with consequences for patient prognosis.

## Conclusion

**Fig. 5:**
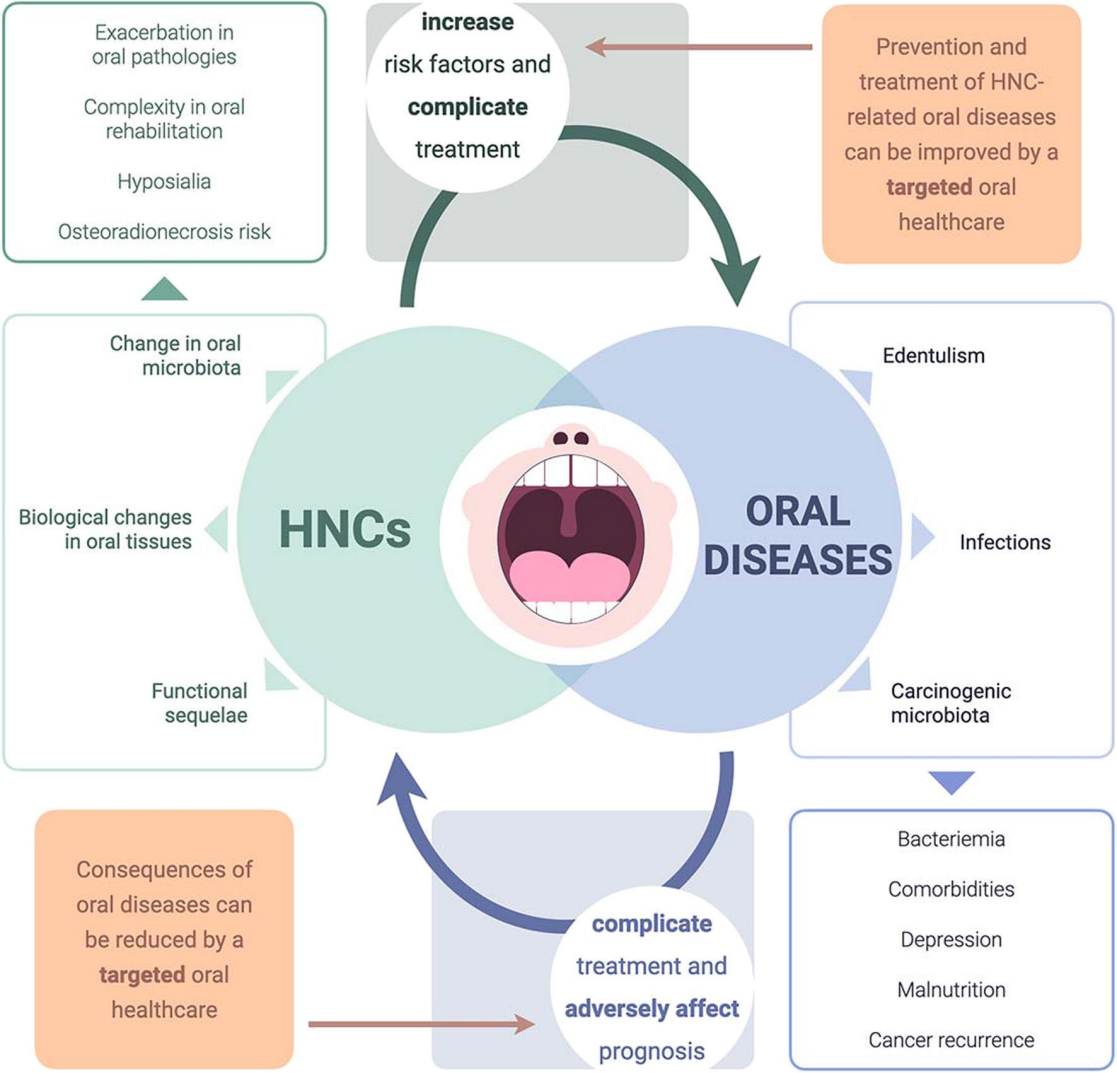
Interrelationships between oral health and HNC

**Fig. 6:**
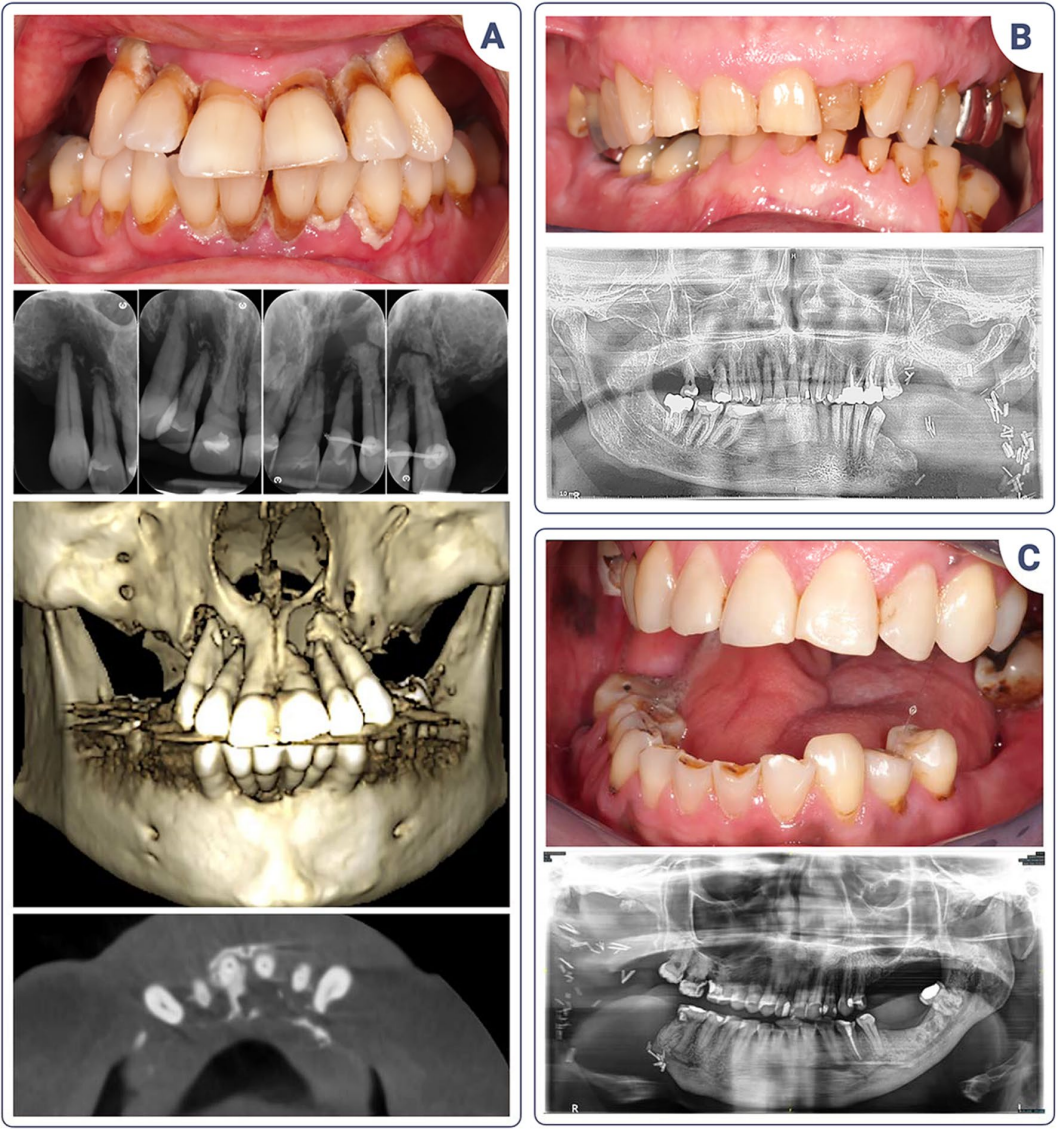
Patients’ voice. **A** Patient with a history of a well-differentiated carcinoma of the soft palate with posterior extension treated with radiochemotherapy (2012). The patient consulted after 10 years of erratic dental care. She arrived at our specialized consultation with advanced, untreated periodontitis associated with extensive ORN of the maxilla requiring inferior maxillectomy. The links between the two conditions were not determined. The patient had consulted five different practitioners who were unable to provide a solution for her painful teeth presenting severe mobility. She felt “abandoned” and was in considerable psychological distress. **B** Patient in remission from a squamous cell carcinoma of the left lingual junction area treated with surgery and radiochemotherapy (2018). At the first consultation, the patient broke down in tears when he was informed that a specialized team was going to take charge of him. Apart from his medical team, he had never met a dentist familiar with HNCs and thought he was in a unique situation that no one knew about. **C** Patient with a history of a right posterior pelvi-mandibular cancer treated with surgery and radiochemotherapy. She had a limited mouth opening and had seen four dentists in private practices who were unable to treat her. In the absence of any viable solution, the clinical situation worsened over the years, with recurrent infectious episodes due to tooth 46, which was necrotic. This dental infection caused an extraoral fistula which was treated by extracting the causal tooth under general anesthesia. She broke down and cried profusely when the team of specialists took charge of her case because she thought she would never find a solution

With over 660,000 new cases of HNCs every year [84] and over 3.5 billion people affected by oral pathologies [10], the oral health of HNC patients cannot be ignored. Indeed, this study highlights the many potential links between oral health and HNC treatment and prognosis (Fig. 5). Moreover, the frequent functional sequelae of previous treatments often require an adaptation of the treatment environment: appropriate psychological approach, short sessions, seated or semi-seated position, prevention of choking, mouth opening limitation, etc. All the specific features described in this article are therefore part of an overall challenge for the dentist. Besides the fact that there are few specialized facilities, the specific oral and dental characteristics of HNC patients are often poorly known by dentists in private practice. As a result, many HNC patients receive no or inadequate care, with substantial consequences on their quality of life, their health, and more generally health economics (Fig. 6).

By recognizing the specific challenges faced by HNC patients in maintaining oral health, healthcare professionals can strive for improved patient outcomes and enhanced quality of life. In contrast to the current race for diagnostic and therapeutic innovations, this work proposes to optimize a dimension (oral healthcare) that has been well described for decades, through communication and knowledge transfer. It will allow to explore the intricacies and advantages of a targeted and consistent oral healthcare management.

## Data Availability

No datasets were generated or analyzed during the current study.
